# Persistent Pulmonary Hypertension of the Newborn: A Practical Review for UK Level 2 Local Neonatal Units

**DOI:** 10.7759/cureus.116003

**Published:** 2026-09-09

**Authors:** Elnazeer Mohamed

**Affiliations:** 1 Pediatrics, East Surrey Hospital, Redhill, GBR

**Keywords:** extracorporeal membrane oxygenation, inhaled nitric oxide, meconium aspiration syndrome, neonatal echocardiography, pphn

## Abstract

In persistent pulmonary hypertension of the newborn (PPHN), pulmonary vascular resistance fails to fall after birth, resulting in right-to-left shunting and hypoxemia out of proportion to the underlying lung disease. Inhaled nitric oxide (iNO) is generally provided by tertiary neonatal intensive care units and neonatal transport teams, while extracorporeal membrane oxygenation (ECMO) is confined to a small number of designated centers. Most affected infants are therefore first recognized and stabilized in a level 2 local neonatal unit, which can provide intubation, ventilation, arterial access, and circulatory support but does not routinely provide iNO or ECMO. This narrative review provides a descriptive synthesis of current guidance rather than a systematic evidence synthesis. It focuses on what a level 2 team can do during the first one to two hours and while awaiting the transport team. It draws on four publicly available UK guidelines, produced by two regional neonatal networks, an NHS trust, and a neonatal transport service, alongside international guidance, neonatal transport literature, and underlying trial evidence. The four are appraised against the domains of the Appraisal of Guidelines for Research and Evaluation II (AGREE II) instrument. None reports a search strategy, grades its recommendations, or declares a conflict of interest, so all four are treated here as consensus documents rather than evidence-based guidelines. The review addresses recognition and the pre- to post-ductal saturation gap, accurate calculation of the oxygenation index in both millimeters of mercury and kilopascals, ventilation and lung volume, oxygenation, carbon dioxide and pH, circulation and metabolic control, sedation, and escalation and transfer. Five escalation decisions are distinguished: contacting the neonatal transport service, discussing the infant with the ECMO center, determining the transfer destination, assessing ECMO eligibility, and initiating ECMO. The level 2 unit is responsible for recognition, stabilization, and contacting the transport service. The discussion with the ECMO center and the choice of destination are led by the transport service and the tertiary unit, and ECMO eligibility and initiation are decisions for the receiving ECMO center. The oxygenation index prompting contact with the ECMO center is therefore not an ECMO initiation criterion. The review also identifies that the numerical ECMO initiation threshold commonly attributed to the Extracorporeal Life Support Organization derives from a 2017 supplement rather than the current guideline, which states the criterion without a number. The four differ across six clinically relevant thresholds, while UK practice diverges from international recommendations most clearly over sildenafil. The review concludes with recommendations for locally agreed thresholds, reliable oxygenation-index calculation, training and imaging access, and shared audit and research. Effective early management depends on timely recognition, optimization of lung and systemic physiology, accurate severity assessment, and early communication with the transport and tertiary teams, all of which lie within the capability of a level 2 unit.

## Introduction and background

Persistent pulmonary hypertension of the newborn (PPHN) occurs when the pulmonary vascular resistance (PVR) fails to fall after birth. Pulmonary pressures remain at or above systemic pressures, blood shunts right to left across the arterial duct and the foramen ovale, and the infant becomes hypoxemic out of proportion to any parenchymal lung disease [1,2]. In many infants the condition is a functional disorder of circulatory transition, not a structural lesion, arising when a normally formed pulmonary vasculature fails to relax. This is not true of every case: the vasculature may instead be abnormally remodeled, or the pulmonary vascular bed may be reduced in cross-sectional area, as it is in the pulmonary hypoplasia that accompanies congenital diaphragmatic hernia, prolonged oligohydramnios, and congenital cystic adenomatoid malformation [3,4]. PPHN is therefore a syndrome, not a diagnosis in its own right. The term describes a physiological state: sustained elevation of PVR with extrapulmonary right-to-left shunting, arising from several different underlying conditions; identifying the cause matters because those conditions require different treatment alongside the same supportive measures [1]. PPHN is not an alternative to parenchymal lung disease. The diagnosis rests in part on excluding severe parenchymal disease and cyanotic congenital heart disease, but the trust guideline is explicit that PPHN may coexist with significant parenchymal lung disease, and where it does, both must be treated [3].

One network guideline states that PPHN is thought to occur in around two per 1000 live births [1]. A second regional guideline states that severe PPHN is believed to affect up to two to six per 1000 live births and to complicate the course of about one in 10 admissions to neonatal intensive care [5]. The two estimates are inconsistent, and both are quoted as stated. PPHN is primarily a disorder of term and late preterm infants, although it can occur at any gestation, and up to one in 10 cases is idiopathic [1,5].

The reason this matters to a level 2 team is one of geography. Inhaled nitric oxide (iNO) is held in tertiary neonatal intensive care units and is carried by the regional neonatal transport teams, which are usually the route by which it reaches an infant outside a tertiary center [1,5]. Extracorporeal membrane oxygenation (ECMO) is available in a small number of designated centers. The infant, however, almost always presents to the local neonatal unit nearest the place of birth. UK network guidance is explicit that the first task belongs to the referring hospital and that the case should be discussed with the regional neonatal transport service and the tertiary unit at the earliest possible opportunity [1,5]. The European Standards of Care for Newborn Health make the same point as a formal standard: management of PPHN in a specialized center improves mortality and morbidity, and every unit should hold a guideline setting out the indications for transfer [6].

This review is written for the level 2 local neonatal unit. UK neonatal services are organized into three tiers, and the nomenclature used by operational delivery networks across NHS England, with which NHS Scotland is aligned, distinguishes the neonatal intensive care unit from the local neonatal unit and the special care unit [7]. The guidelines cited here describe this setting variously as “non-tertiary” or as “the referring hospital”; for consistency, the designation “level 2” is used throughout. Such a unit has the ventilation, arterial access, imaging, and staffing to intubate and stabilize a term infant with severe hypoxemia but holds neither iNO nor ECMO, which the European standard states should be delivered only in designated centers [6]. UK guidance is written on that assumption. One network guideline is aimed explicitly at initial stabilization before transfer, with iNO started when the transport team arrives [1], and a second regional guideline addresses itself directly to “a unit that does not routinely use iNO” [5].

Two features of the UK setting make an account written specifically for this level worthwhile. Access to specialist therapy is uneven, so the interventions that matter most in the first hours are those a level 2 unit already holds. Regional guidance also varies. As this review shows, the four current UK guidelines examined differ on six clinically relevant thresholds. These are the saturation gap that counts as significant, the blood pressure target, the hemoglobin threshold, the carbon dioxide target, the oxygenation index at which iNO is started, and the oxygenation index at which the ECMO center is contacted. These differences are not resolved by NICE, which excluded PPHN from the scope of its neonatal guidance [8]. The problem is not confined to the UK: a large North American center published its own cross-disciplinary PPHN guideline precisely because no universal contemporary guideline existed and reached the same conclusion that the evidence base is too thin to settle the operational figures [9]. The review is therefore organized around what such a unit can deliver, when it should call, and what it should do while waiting. Management belonging to the transport team or the receiving center is set out separately and briefly, because the referring team needs to understand that conversation, not to carry it out.

The question this review asks is therefore a narrow one: what should a level 2 team do in the first one to two hours, and when should it hand over? Where the four guidelines differ, that difference is stated rather than smoothed over. A clinician using this review must ultimately be guided by their own network guideline.

Five decisions are involved in escalation, and they are kept separate throughout this review because they arise at different times, are taken by different people, and are triggered by different figures. Calling the regional neonatal transport service is a request for advice and does not commit the infant to being moved. Discussing the infant with the ECMO center is a consultation, not a decision to cannulate. Transferring to an ECMO-capable center is a question of destination, decided by whether the infant may meet eligibility. Eligibility is a separate judgement, made at the receiving center against published criteria for gestation, weight, reversibility of the lung disease, and contraindications. Initiation is the decision to cannulate, taken at the ECMO center. Only the first belongs wholly to the level 2 team, and the oxygenation index that justifies a telephone call is not an ECMO initiation criterion.

Methodology

This is a narrative review. Its objective is a descriptive synthesis of current UK and international guidance for the level 2 setting, together with the trial evidence on which that guidance rests. It was not conducted as a systematic review and does not claim to be one. The search had three strands, set out below so that it can be repeated.

Bibliographic Search

MEDLINE (via PubMed), Embase, and the Cochrane Library were searched through the NHS Knowledge and Library Hub on 10 June 2026. The search combined ("persistent pulmonary hypertension of the newborn" OR "PPHN" OR "persistent foetal circulation") AND ("meconium aspiration syndrome" OR "oxygenation index" OR "inhaled nitric oxide" OR "milrinone" OR "sildenafil" OR "neonatal transport" OR "functional echocardiography" OR "neonatologist performed echocardiography"), as title, abstract, and keyword terms, with the corresponding subject headings where the database supported them. Records were limited to English-language publications from the preceding 10 years. Reference lists of retrieved articles were hand-searched, and priority was given to systematic reviews, randomized controlled trials, and clinically relevant neonatal reviews. Records outside that window were retained where they remain the current version of a guideline, the most recent systematic review on a question, or the primary description of a rare disorder.

Guideline Search

Publicly available UK guidance was sought from the open web, the guideline libraries of NHS neonatal operational delivery networks, NHS trusts and neonatal transport services, and the National Institute for Health and Care Excellence (NICE) website. Documents were eligible if they were publicly accessible without subscription, carried an identifiable issuing organization and a date or version number, and addressed the management of PPHN or iNO in the newborn; local proformas were excluded. Four were then selected purposively to span the types of organizations that issue such guidance and different parts of the UK. These were a regional neonatal operational delivery network guideline [1], an NHS trust guideline [3], a transport service guideline [10] and a second regional network guideline from Scotland [5]. Transport service configuration varies across the UK; the third is issued by a service that describes itself as providing pediatric intensive care retrieval, and its guideline addresses PPHN in the newborn. Throughout this review, references to the guidelines examined and to UK guidance where no other source is named mean these four documents and not UK practice as a whole. National guidance from NICE was searched separately and is cited where it applies [8,11,12].

International Guidance

Two documents are used for comparison throughout: the American Heart Association and American Thoracic Society guidelines on pediatric pulmonary hypertension, which grade their recommendations [13], and the European Standards of Care for Newborn Health [6]. Extracorporeal Life Support Organization (ELSO) criteria are cited directly from the ELSO documents themselves: the current guideline for neonatal respiratory failure [14] and the earlier supplement from which the numerical initiation criterion derives [15].

Appraisal

The four UK guidelines were appraised against the domains of the Appraisal of Guidelines for Research and Evaluation II (AGREE II) instrument: scope and purpose, stakeholder involvement, rigor of development, clarity of presentation, applicability, and editorial independence, together with version and currency. It is a structured appraisal, not a formal AGREE II assessment, for the reason given in the Limitations. Each guideline is also cited with its version number and review or expiry date so that a reader can verify the version used. This review reports what the cited guidelines say and issues no doses or thresholds of its own; statements attributed to a named guideline are descriptive, and the author's own recommendations are confined to the Recommendations section.

Synthesis and Prioritization

Sources were not pooled, and where they conflict, no attempt is made to reconcile them. Four rules were applied consistently. First, claims about whether a treatment works are cited to systematic reviews or randomized trials, not to a guideline repeating them. Second, operational thresholds and targets are taken from the guidelines that issue them, because these are matters of local policy for which no primary study provides an answer; where guidelines give different figures, each is reported with its own source. Third, where a document cites a criterion from another body, the primary document is retrieved and cited instead, which was applied to the ELSO criteria, where the secondary citation proved inaccurate. Fourth, where UK practice diverges from international guidance, both positions are stated with their sources and the reason for the divergence given rather than one being presented as correct. Divergences of this kind are tabulated later in this review.

Evidence Strength Framework

To make the basis of each figure visible, sources are described throughout in five categories. Trial evidence means a systematic review or randomized controlled trial. A graded guideline recommendation is one to which an international guideline assigns a class and level. National guidance means guidance issued after formal evidence review by a national body. Guideline consensus means regional network, NHS trust, or transport service guidance that states no grading. Narrative review means a peer-reviewed review without a systematic method. These categories are used to classify each threshold reported and to distinguish three kinds of statement throughout: recommendations resting on trial evidence or on a graded international recommendation; figures based on local or regional consensus, as all four UK documents were found to be; and the authors’ own practical suggestions, which appear only in the Recommendations section and are not cited to any guideline.

Doses and Thresholds

Drug doses are quoted as the cited documents state them, and each was then compared with the British National Formulary for Children [16]. Where a guideline dose differs from the formulary, that difference is stated in the text rather than resolved, on the same principle applied to divergent thresholds. Physiological targets and escalation thresholds are reported with their individual sources and with the basis on which each rests, and national guidance is cited where it exists. Contraindications to extracorporeal support were taken from the primary ELSO documents, not from any secondary account of them. Monitoring requirements, including methemoglobin and inspired nitrogen dioxide during iNO therapy, are quoted from the guidelines that specify them, with the differing figures reported separately. Every dose, target, and criterion must still be checked against the reader’s own formulary and local policy before use.

## Review

Pathophysiology in brief

Before turning to the physiology, the four UK guidelines examined throughout this review are set out in Table 1, with the version, approval date, and review date of each, so that a reader can confirm which edition is being quoted.

**Table 1:** Structured appraisal of the four UK guidelines examined, informed by AGREE II domains. The table records verifiable characteristics of each document against the domains of the AGREE II instrument. It is a structured appraisal, not a formal AGREE II assessment: scoring the instrument requires more than one appraiser, and the appraisal reported here was made by a single author, which is stated as a limitation. Three findings are common to all four documents. None reports a search strategy, none grades the strength of its recommendations, and none carries a conflict-of-interest statement or declares the funding of the guideline’s development. On the AGREE II criteria, therefore, all four are characterized here as local or regional consensus documents rather than evidence-based guidelines. Scope and applicability differ more between the four documents than provenance does. Compiled by the author from the guideline documents themselves. AGREE II, Appraisal of Guidelines for Research and Evaluation II; iNO, inhaled nitric oxide; ODN, operational delivery network; PPHN, persistent pulmonary hypertension of the newborn

Appraisal dimension	Regional neonatal ODN [1]	NHS trust [3]	Transport service [10]	Regional network (Scotland) [5]
Type of issuing organization	Regional neonatal operational delivery network	NHS trust	Pediatric intensive care retrieval service, as the document describes itself	Regional network, Scotland
Stated scope and purpose (AGREE II domain 1)	Coordinated initial stabilization of term and near-term PPHN in non-tertiary settings, and ongoing guidance for the transport service and tertiary centers	Guidance on the acute clinical management of neonatal PPHN	Assessment, immediate and ongoing management, and transport considerations in PPHN	Safe and consistent administration of inhaled nitric oxide in the neonatal population, stated as based on current best practice and evidence
Intended audience	Not stated explicitly; organized by care setting	The issuing trust only; the community trust and integrated care board are excluded	All employees of the transport service	All medical, nursing, and midwifery staff administering iNO in the region
Stakeholder involvement (domain 2)	Three named consultant neonatologists; no multidisciplinary or parent involvement stated	Named consultant author and named reviewer; approved by the trust neonatal audit and guidelines group, with consultation recorded	Named consultant author and named document owner; no wider involvement stated	Single named consultant author; multidisciplinary audience but no multidisciplinary authorship stated
Rigor of development (domain 3)	Reference list of 10 sources; no search strategy, no evidence grading, no external review stated	Eleven related documents listed, including textbooks, primary trials, and other units' guidelines; no search strategy, no evidence grading	No reference list, no search strategy, no evidence grading	Reference list of 17 sources; no search strategy, no evidence grading
Clarity of presentation (domain 4)	Flow chart, tabulated stabilization measures, and boxed drug protocols	Summary box, structured sections, and an echocardiography appendix	Short, structured document in bulleted form	Structured web-based guideline
Applicability to a level 2 unit (domain 5)	High; written explicitly for the non-tertiary setting	High; written for a referring unit that escalates to a transport service	Indirect; written for transport staff, though its stabilization targets apply at the bedside	Partial; the scope is iNO administration rather than PPHN management as a whole
Editorial independence (domain 6)	No conflict-of-interest or funding statement	No conflict-of-interest statement; governance and approval route documented	No conflict-of-interest statement; the page footer states the funding and accountability of the service, but not of the guideline	No conflict-of-interest or funding statement
Contribution to the pathway described here	The escalation structure: the trigger for calling the transport service, the threshold for the ECMO discussion, the stepwise circulatory protocol, and most physiological targets	Recognition criteria, the ECMO inclusion criteria, the alternative carbon dioxide and blood pressure targets, and the sedation and muscle relaxant regimen	The index expressed in millimeters of mercury, the ventilation strategy where high-frequency oscillation is unavailable, transport preparation, and therapeutic magnesium	The indication for iNO, the weaning schedule and methemoglobin monitoring, the hemoglobin target before iNO, and the preterm exception after prolonged rupture of membranes
Version and currency	Version 2, reviewed December 2024, next review December 2029; current	Version 4.0, approved August 2025, valid to August 2028; current	Version 4, active January 2020, next review January 2022; past its stated review date	Not version-numbered; reviewed April 26, 2024, next review April 26, 2027; current

In utero, the pulmonary circulation is a high-resistance, low-flow system that receives only a small fraction of the combined ventricular output [2,17]. At birth, lung aeration and the rise in arterial oxygen tension are the principal stimuli for pulmonary vasodilation, causing PVR to fall sharply [2,5]. Systemic vascular resistance rises with cord clamping, flow across the ductus reverses, and the fetal channels close over the following hours [5].

In PPHN, this transition is attenuated or reversed. Endothelial dysfunction impairs nitric oxide-cyclic guanosine monophosphate signaling, while hypoxia and acidosis directly contract pulmonary vascular smooth muscle [4,17].

The underlying mechanism varies with the cause. Acute vasoconstriction predominates in meconium aspiration syndrome (MAS), asphyxia, sepsis, and respiratory distress syndrome; vascular remodeling predominates after chronic intrauterine hypoxia, antenatal ductal closure, and congenital diaphragmatic hernia. Intravascular obstruction occurs in polycythemia, while pulmonary hypoplasia reduces the cross-sectional area of the vascular bed [2,4,5]. This distinction matters outside tertiary care. Raised pre-capillary vascular resistance is the commonest phenotype and is the most responsive to recruitment, oxygenation, and correction of metabolic abnormalities [17]. Hypoplastic disease is less responsive to these measures and is more likely to require ECMO [4,17].

Recognizing PPHN in a level 2 unit

Recognition is framed around a persistently high oxygen requirement in a term or near-term infant. One trust guideline recommends considering PPHN beyond 35 weeks’ gestation when there is a sustained oxygen requirement above 50% or an arterial partial pressure of oxygen (PaO₂) below 8 kPa, together with a pre- to post-ductal saturation difference above 4% and respiratory distress [3]. However, the most important clinical feature is difficulty maintaining adequate oxygenation despite escalating respiratory support [3].

A pre- to post-ductal saturation difference of approximately 5-10% supports right-to-left ductal shunting but is not diagnostic of PPHN [6]. The relationship between ductal shunting and the saturation gap is illustrated in Figure 1.

**Figure 1:**
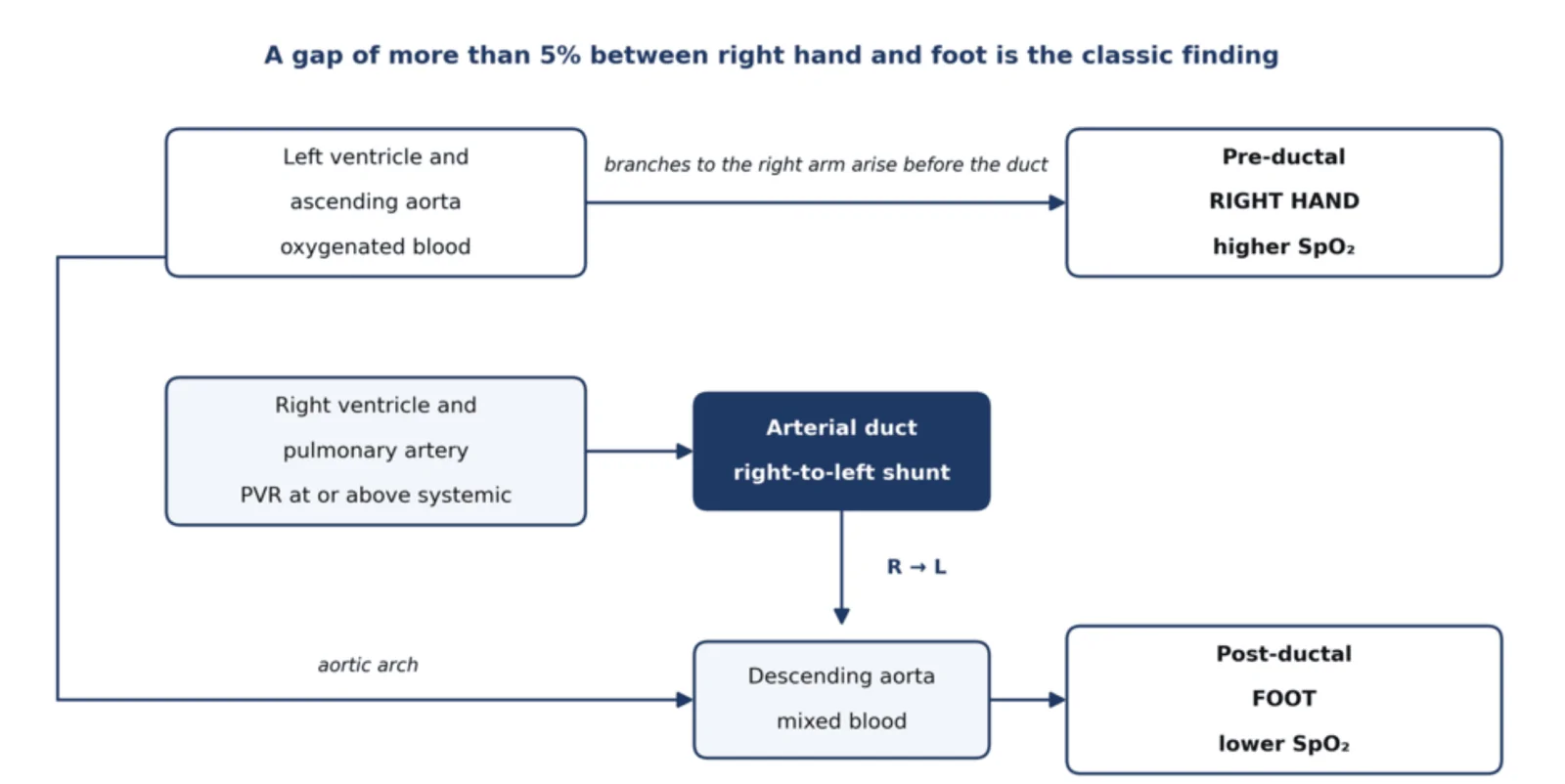
Pre- to post-ductal saturation difference supporting right-to-left ductal shunting. The gap may be absent when right ventricular output is low, the duct is small or closed, shunting is predominantly atrial, or flow across the duct is balanced and bidirectional. It is also absent when hypoxemia is driven mainly by shunting within the lung, as in MAS, respiratory distress syndrome, or pneumonia. Right-to-left ductal shunting can produce lower post-ductal (foot) saturations than pre-ductal (right-hand) saturations. The size of difference each document treats as significant varies, and the figures are given with their sources in Table 2. The circumstances in which the gap may be absent, and in which it therefore does not exclude PPHN, are annotated on the figure [1,3]. L, left; MAS, meconium aspiration syndrome; PVR, pulmonary vascular resistance; R, right; SpO₂, peripheral oxygen saturation

**Table 2:** Contested thresholds and the five escalation decisions in PPHN, one row per source. The table is confined to thresholds on which the documents examined differ and to criteria defining the five escalation decisions. Uncontested stabilization targets are presented in the text and Figure 2 rather than repeated here. Documents with differing figures are kept in separate rows. Where documents state the same figure in the same terms, they share a row, and both are cited. The fifth column gives the issuing document in abbreviated form with its version or year; full provenance is provided in Table 1. The sixth column states the basis for each figure using the evidence categories defined in the review methodology. Three labelled blocks distinguish treatment goals from escalation thresholds and decisions. Section A presents contested physiological targets: these are treatment goals and are not, in themselves, reasons to escalate. Section B presents contested diagnostic thresholds. Section C presents escalation triggers and the five numbered decisions. The table reports what each document states; it does not recommend one figure over another. Local network policy takes precedence throughout. AHA/ATS, American Heart Association and American Thoracic Society; ECMO, extracorporeal membrane oxygenation; ELSO, Extracorporeal Life Support Organization; iNO, inhaled nitric oxide; NICE, National Institute for Health and Care Excellence; ODN, operational delivery network; OI, oxygenation index; PaCO₂, arterial partial pressure of carbon dioxide; PaO₂, arterial partial pressure of oxygen; SpO₂, peripheral oxygen saturation

Domain	Parameter, threshold, or target	Population	Intended action	Source document and version	Basis
A. Physiological targets - treatment goals, not triggers for escalation					
Oxygenation	Pre-ductal SpO₂ 95-98%, aiming to narrow the pre- to post-ductal gap to below 5%	Term/near-term	Titrate inspired oxygen.	Regional ODN, v2 (2024) [1]	Guideline consensus
Oxygenation	PaO₂ above 8 kPa, preferably 8-10 kPa	Term/near-term	Titrate inspired oxygen.	Transport service, v4 (2020) [10]	Guideline consensus
Carbon dioxide and pH	PaCO₂ 4.5-5.5 kPa with pH 7.35-7.45	Term/near-term	Set ventilation	Regional ODN, v2 (2024) [1]	Guideline consensus
Carbon dioxide and pH	PaCO₂ 5-7 kPa if stable, with pH above 7.35	Term/near-term	Set ventilation	NHS Trust, v4.0 (2025) [3]	Guideline consensus
Blood pressure	Mean blood pressure above 45-50 mmHg	Term/near-term	Circulatory support	Regional ODN, v2 (2024) [1]	Guideline consensus
Blood pressure	Mean arterial pressure above 60 mmHg, or higher if calculated right ventricular pressure exceeds this	Term	Circulatory support	NHS Trust, v4.0 (2025) [3]	Guideline consensus
Blood pressure	Mean arterial pressure above the estimated pulmonary pressure, which may require above 60 mmHg	Term/near-term	Circulatory support	Transport service, v4 (2020) [10]	Guideline consensus
Blood pressure	Upper end of the normal range for gestation, about 10 mmHg above the gestational age in weeks	Term/near-term	Circulatory support	Regional network, reviewed 2024 [5]	Guideline consensus
Blood pressure	Do not treat hypotension on a blood pressure threshold alone; aim to improve perfusion.	Preterm (extrapolated)	Circulatory support	NG124 (2019) [11]	National guidance
Hemoglobin	Hemoglobin above 140 g/L	Term/near-term	Transfusion	Regional ODN, v2 (2024) [1]	Guideline consensus
Hemoglobin	Hemoglobin above 120 g/L	Term/near-term	Optimize before iNO is started.	Regional network, reviewed 2024 [5]	Guideline consensus
B. Diagnostic thresholds - what supports or grades the diagnosis					
Saturation gap	Pre- to post-ductal gap above 5% is the classic finding; 5-10% is suggestive.	Term/near-term	Support the diagnosis.	Regional ODN, v2 (2024) [1]	Guideline consensus
Saturation gap	Pre- to post-ductal gap above 10% marks significant right-to-left shunting.	Beyond 34 weeks	Support the diagnosis.	Regional network, reviewed 2024 [5]	Guideline consensus
C. Escalation triggers and decisions - when to act and whom to call					
Starting iNO	OI above 15-20, or above 60% oxygen where the OI cannot be calculated	Term/near-term	Start iNO in practice on arrival of the transport team.	Regional ODN, v2 (2024) [1]	Guideline consensus
Starting iNO	OI above 15, or a saturation gap above 5% without congenital heart disease	Term/near-term	Start iNO.	NHS trust, v4.0 (2025) and neonatal transport service guideline, version 4 (2020), in the same terms [3,10]	Guideline consensus
Starting iNO	Hypoxemic respiratory failure not responding to optimal ventilation, without a numerical threshold	Beyond 34 weeks	Start iNO.	Regional network, reviewed 2024 [5]	Guideline consensus
Starting iNO	OI above 25	Term/near-term	Start iNO.	AHA/ATS guideline (2015) [13]	Graded recommendation (Class I, Level A)
Decision 1: call the transport service.	OI 15-20 on optimized conventional ventilation, or an oxygen requirement above 60% where the OI cannot be calculated	Term/near-term	Telephone the regional neonatal transport service for advice; does not commit the infant to being moved.	Regional ODN, v2 (2024) [1]	Guideline consensus
Decision 2: discuss with the ECMO center	OI above 30, or failure to respond to treatment	Term/near-term	Conference call with the ECMO center; a consultation, not a decision to cannulate	Regional ODN, v2 (2024) [1]	Guideline consensus
Decision 2: discuss with the ECMO center	OI above 25	Term/near-term	Consider the ECMO referral and begin the conversation.	NHS Trust, v4.0 (2025) [3]	Guideline consensus
Decision 2: discuss with the ECMO center	Severe pulmonary hypertension or hypoxemia refractory to iNO and to optimization of respiratory and cardiac function; an OI above 40 is given as an indication to consider referral.	Term/near-term	Refer to an ECMO center.	AHA/ATS guideline (2015) [13]	Graded recommendation (Class I, Level A)
Decision 3: transfer to an ECMO-capable center	No separate numerical threshold; determined by whether the infant may meet eligibility and settled before the transfer is arranged rather than after	Term/late preterm	Choose the destination center before the infant is moved	Regional ODN, v2 (2024) [1]	Guideline consensus
Decision 4: eligibility	Gestation below 34 weeks, weight below 2 kg, a known non-reversible condition and significant intracranial injury, each an exclusion on its own	Term/late preterm	Establish whether ECMO is available to this infant.	Regional ODN, v2 (2024) [1]	Guideline consensus
Decision 4: eligibility	Gestation of 34 weeks or a weight of 2 kg, either sufficient; OI above 25, the same figure that the document gives for beginning the ECMO conversation; reversible lung disease, defined as fewer than 10 days of high-pressure ventilation; no lethal congenital abnormality	Term/late preterm	Establish whether ECMO is available to this infant.	NHS Trust, v4.0 (2025) [3]	Guideline consensus
Decision 4: eligibility	High likelihood of mortality with a potentially reversible etiology; no lethal chromosomal disorder or other lethal anomaly, severe brain damage, uncontrollable bleeding, significant intraventricular hemorrhage, or vessels too small to cannulate. Weight below 2 kg and gestational age below 34 weeks are relative contraindications.	Term/late preterm	Establish whether ECMO is available to this infant.	ELSO guideline (2020) [14]	Guideline consensus
Decision 4: eligibility	Mechanical ventilation beyond 10 to 14 days, given as a further relative contraindication	Term/late preterm	Establish whether ECMO is available to this infant.	ELSO supplement, v1.4 (2017) [15]	Guideline consensus
Decision 5: cannulation and initiation	Oxygenation index with sustained elevation and no improvement, stated without a numerical threshold, alongside inadequate tissue oxygen delivery despite maximal therapy	Term/late preterm	Initiate ECMO; a decision taken at the ECMO center	ELSO guideline (2020) [14]	Guideline consensus
Decision 5: cannulation and initiation	OI above 40 sustained for more than four hours, calculated from the post-ductal PaO₂, alongside refractory clinical features	Term/late preterm	Initiate ECMO; a decision taken at the ECMO center	ELSO supplement, v1.4 (2017) [15]	Guideline consensus; wording as quoted

**Figure 2:**
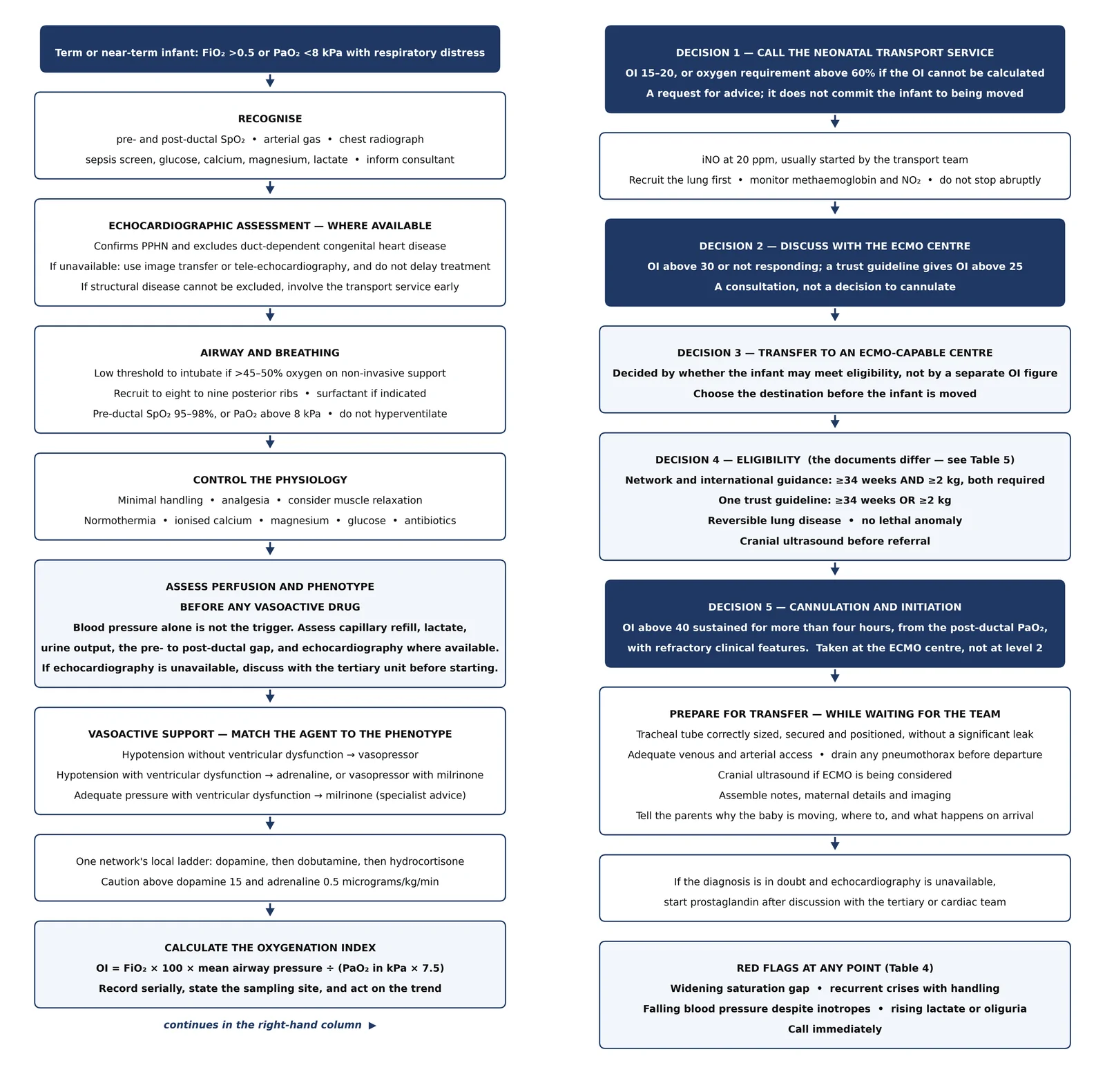
Initial management during the first one to two hours in a UK level 2 local neonatal unit. The sequence is designed for a unit that holds neither iNO nor ECMO. Shading marks ownership: white and pale boxes are delivered by the level 2 unit; navy boxes are decisions taken with, or by, the transport service, the tertiary unit, or the ECMO center. Echocardiographic assessment is shown as an explicit step, with the alternative route where it is not available locally. The five escalation decisions are shown separately because they are triggered by different thresholds and taken by different teams. Assessment of perfusion and hemodynamic phenotype precedes vasoactive treatment, with the agent matched to the phenotype rather than applied as a fixed sequence. The stepwise dopamine, dobutamine, and hydrocortisone protocol is shown separately as one network’s local pathway [1]. The thresholds in each decision box are those reported, with their sources, in Table 2. ECMO, extracorporeal membrane oxygenation; FiO₂, fractional inspired oxygen concentration; iNO, inhaled nitric oxide; NO₂, nitrogen dioxide; OI, oxygenation index; PaO₂, arterial partial pressure of oxygen; PPHN, persistent pulmonary hypertension of the newborn; SpO₂, peripheral oxygen saturation Compiled by the author from [1,3,5,10,14,15].

The UK guidelines examined are clear that the absence of a saturation gap does not exclude PPHN [1,3]. The gap may be absent when right ventricular output is low, the ductus is small or closed, or shunting is predominantly atrial or balanced across both shunts [1,3]. Conversely, a narrowing gap during treatment is a useful bedside sign of improving pulmonary blood flow [1].

The saturation gap reflects shunting across the arterial duct. Hypoxemia is caused predominantly by intrapulmonary shunting, as in significant MAS, respiratory distress syndrome, or pneumonia, therefore, lowers saturation at both sites equally and produces no gap. Equally, when ductal and atrial shunting is left to right, the hypoxemia is primarily intrapulmonary, and the priority is lung recruitment, not pulmonary vasodilation [2]. Because PPHN commonly coexists with parenchymal lung disease [3], uniformly low saturations do not exclude it.

Suspicion should increase in a term or late preterm infant with meconium-stained liquor, perinatal asphyxia, sepsis, particularly group B streptococcal infection, or pneumonia. It should also increase when pulmonary hypoplasia is expected, as in congenital diaphragmatic hernia, oligohydramnios, or prolonged rupture of membranes [3,10]. Polycythemia and hypocalcemia may contribute, while maternal aspirin, non-steroidal anti-inflammatory drugs, and selective serotonin reuptake inhibitors are recognized associations [3,10]. Pulmonary hypertension can also occur in preterm infants, in whom the evidence for iNO differs from that in term infants and is considered later [5,13]. Idiopathic disease may be particularly difficult to recognize because the chest radiograph can appear deceptively clear despite severe hypoxemia [1,10].

Congenital heart disease is the critical differential diagnosis. Where echocardiography is unavailable, features favoring a cardiac cause include cardiomegaly, weak pulses, an active precordium, a murmur, and little or no response to oxygen. UK network guidance notes that distinguishing PPHN from duct-dependent pulmonary lesions can be difficult; where doubt remains, a prostaglandin infusion is generally the safest option during transfer [1].

The Hyperoxia Test: A Point of Disagreement

The guidelines examined differ on the role of the hyperoxia test, and this uncertainty is relevant to level 2 units. One network guideline states that the test is no longer considered useful and may cause harm through over-oxygenation [1]. A current trust guideline retains a trial of 100% oxygen when two-dimensional echocardiography is unavailable, while acknowledging that severe PPHN may produce a similar response to cyanotic congenital heart disease [3].

The disagreement is therefore about practice rather than the test's limitation: both documents recognize that a rise in PaO₂ does not exclude a cardiac cause. International guidance avoids the test altogether and recommends echocardiography to establish the diagnosis [13].

Where echocardiography or image transfer can be arranged promptly, this is the preferred diagnostic approach. If the hyperoxia test is used because echocardiography is unavailable, its result should be interpreted alongside the clinical examination and pre- to post-ductal saturation difference. It should not be used alone to exclude duct-dependent congenital heart disease or to withhold prostaglandin [1,3].

Imaging and Echocardiography Without On-Site Expertise

Chest radiography helps exclude other pathology, such as pneumothorax; identify causes such as MAS; confirm tube and line position; and guide lung inflation [1,3]. Bedside lung ultrasound can identify parenchymal abnormalities, including consolidation and atelectasis, and may help distinguish parenchymal lung disease from predominantly vascular presentations when interpreted alongside the clinical and echocardiographic findings [18,19].

Echocardiography supports the diagnosis of PPHN by demonstrating a tricuspid regurgitant jet, septal flattening or bowing, right ventricular size and function, and the direction of ductal and atrial shunting [3,10]. Echocardiography is also essential for excluding structural or duct-dependent congenital heart disease [10]. UK guidance is clear that when echocardiography is not readily available, waiting for it should not delay treatment; if the clinical picture is consistent with PPHN, treatment should proceed [1].

Units without on-site expertise should use image transfer to the regional pediatric cardiology service and tele-echocardiography links, which the trust guideline describes as routine practice [3]. This should be supported by recognized European competency frameworks for neonatologist-performed echocardiography and point-of-care ultrasound [20,21]. However, access to 24-hour echocardiography by a neonatologist or cardiologist varies between level 2 units. Where structural congenital heart disease is suspected but cannot be excluded locally, particularly after prostaglandin has been started, this should prompt early involvement of the transport service. Definitive exclusion of congenital heart disease rests with pediatric cardiology, not the referring unit [10].

Quantifying Severity: The Oxygenation Index in Kilopascals

The oxygenation index (OI) is an important measure of hypoxemic respiratory failure and is used in UK guidance to inform escalation and referral. It is conventionally written with the arterial oxygen tension in millimeters of mercury, and one UK transport service guideline states it in that form [10]:

$\mathrm{OI} = \frac{\text{mean airway pressure (cmH}_2\mathrm{O)} \times \mathrm{FiO}_2 \times 100}{\mathrm{PaO}_2\text{ (mmHg)}}$

A recognized pitfall is the unit conversion. UK arterial blood gases are reported in kilopascals, so the conventional thresholds cannot be applied to a UK gas without converting; one network guideline spells out the conversion factor and warns explicitly that units must be checked when an online calculator is used [1]. When PaO₂ is reported in kPa, two of the guidelines examined give the index as [1,3]:

$\mathrm{OI} = \frac{\mathrm{FiO}_2 \times 100 \times \text{mean airway pressure (cmH}_2\mathrm{O)}}{\mathrm{PaO}_2\text{ (kPa)} \times 7.5}$

FiO₂ should be expressed as a fraction.

For example, with a mean airway pressure of 14 cmH₂O, an FiO₂ of 1.0 (100%) and a PaO₂ of 5.3 kPa:

\(\mathrm{OI} = \frac{(1.0 \times 100 \times 14)}{(5.3 \times 7.5)} = \frac{1400}{39.75} \approx 35\)

If 5.3 kPa were entered without applying the ×7.5 conversion, the OI would be approximately 264, substantially overestimating the severity of hypoxemia. Both the FiO₂ fraction and PaO₂ unit conversion must therefore be applied correctly.

When OI is calculated from an umbilical arterial catheter, the sample is post-ductal, and UK guidance makes this explicit [1,3]. The sampling site must therefore be stated whenever the index is recorded or handed over, because a pre-ductal and a post-ductal sample in the same infant can give materially different indices, and the ECMO initiation criterion is defined on the post-ductal value [15]. OI should be recorded serially instead of as a single value because the trend helps determine whether the infant is deteriorating or responding to treatment [1]. For descriptive purposes, this review uses OI bands of ≤15, >15-25, >25-40 and >40; these should not be interpreted as universally accepted treatment thresholds [2]. UK targets and action thresholds are summarized in Table 2.

Four different decisions are attached to the index, and they must be kept apart. They do not map one to one onto the five escalation decisions set out earlier: two of those five, the choice of destination and the assessment of eligibility, carry no index figure at all, whereas the decision to start iNO does. The four are, in the order in which they usually arise: specialist consultation, meaning the first telephone call to the transport service; consideration of iNO; contact with the ECMO center; and initiation of extracorporeal support. Each is triggered by a different figure, each is taken by different people, and only the first belongs to the level 2 unit. In the documents examined here the figures run as follows: consultation at an index of 15-20, or an oxygen requirement above 60% where the index cannot be calculated; iNO at an index above 15 in two documents, above 15-20 in a third, and above 25 in the international guideline; contact with the ECMO center at an index above 25 or above 30; and initiation at an index above 40 sustained for more than four hours. Each figure is given with its own source in Table 2.

International guidance recommends iNO for term and near-term infants with PPHN or hypoxemic respiratory failure when the OI exceeds 25 [13]. In the UK, however, this decision belongs to the transport team or tertiary center, not the referring unit. An OI above 40 is an indication to consider referral to an ECMO center [13]. ECMO should not be considered on the OI alone: persistent severe pulmonary hypertension or hypoxemia despite iNO and optimization of respiratory and cardiac function should guide that decision [13]. Across the documents examined, however, thresholds above 25, 30, and 40 are used, and no single figure represents a universal ECMO criterion.

The two ELSO documents for neonatal respiratory failure differ in how precisely they express the initiation criterion. In the 2017 supplement, the criterion is an OI above 40 sustained for more than four hours, calculated from the post-ductal PaO₂ [15]. The current 2020 guideline describes the criterion as sustained elevation of the OI without improvement, without specifying a numerical threshold, alongside inadequate tissue oxygen delivery despite maximal therapy, severe hypoxemic respiratory failure with acute decompensation, and severe pulmonary hypertension with ventricular dysfunction [14]. That numerical threshold therefore derives from the earlier supplement, not the current guideline. Although the European service standard cites the ELSO criteria, it quotes a different figure; the ELSO documents should therefore be consulted directly [6,14,15].

Ventilation

Network guidance advises a low threshold for intubation. One network recommends considering intubation in an infant requiring more than 45-50% oxygen to maintain saturations on non-invasive support [1]. Adequate lung volume is itself a pulmonary vasodilator maneuver because atelectasis increases PVR within minutes; however, overdistension also raises PVR, so lung volume should be optimized, not maximized [2,13]. UK guidance targets expansion to eight or nine posterior ribs on chest radiography while avoiding overexpansion [1,10].

A collapsed lung remains perfused, allowing blood to pass through without adequate oxygen uptake and adding an intrapulmonary shunt to the extrapulmonary shunt. Recruitment therefore addresses both mechanisms. Where shunting is predominantly intrapulmonary, optimizing lung volume with positive end-expiratory pressure and adequate mean airway pressure, with surfactant where indicated, improves oxygenation. Where right-to-left shunting occurs across both the ductus and foramen ovale, indicating raised PVR and extrapulmonary shunting, pulmonary vasodilation becomes the primary treatment once the lung is adequately recruited [2].

Ventilation should be matched to the underlying lung disease. Parenchymal diseases such as MAS or pneumonia generally require adequate positive end-expiratory pressure and volume-targeted ventilation, whereas gentler settings are appropriate when the lungs are clear [1,2]. Where high-frequency oscillatory ventilation is unavailable, transport service guidance suggests conventional ventilation with a positive end-expiratory pressure of 8-10 cmH₂O to improve oxygenation while limiting peak pressures [10]. Routine hyperventilation to induce respiratory alkalosis is not recommended: it has not demonstrated long-term benefit, and hypocapnia may adversely affect the cerebral and pulmonary circulation [3,13].

Oxygen saturation targets differ between the guidelines examined. One network advises pre-ductal saturations of 95-98%, on the basis that lower saturations may potentiate hypoxic pulmonary vasoconstriction, with a pre- to post-ductal gap below 5% as the treatment aim [1]. A transport service guideline expresses the target as a PaO₂ above 8 kPa, preferably 8-10 kPa [10]. Network guidance cautions against hyperoxia because reactive oxygen species can increase pulmonary arterial contractility and directly deactivate nitric oxide [1,2]. Network guidance acknowledges that evidence for oxygen targeting in PPHN remains inconclusive; PaO₂ and OI should therefore be considered together when titrating oxygen [1]. International guidance similarly cautions that extreme hyperoxia, with FiO₂ above 0.6, may be ineffective in the presence of extrapulmonary shunting and may aggravate lung injury [13].

Carbon dioxide and pH targets also vary. One network advises a PaCO₂ of 4.5-5.5 kPa with a pH of 7.35-7.45 [1]. A trust guideline accepts a PaCO₂ of 5-7 kPa in a stable infant provided the pH remains above 7.35 [3]. A transport service guideline advises reducing PaCO₂ to normal while explicitly avoiding hypocapnia [10]. All reject hyperventilation. International guidance provides the rationale: forced alkalosis, whether achieved by hyperventilation or bicarbonate, may transiently improve PaO₂ but has not demonstrated long-term benefit; prolonged alkalosis may also worsen pulmonary vascular tone and reduce cerebral blood flow [13].

Surfactant at 200 mg/kg may be considered when PPHN is associated with significant surfactant deficiency or severe MAS, rather than as routine therapy for all PPHN [1,3]. Neither document names the product, although the figure quoted corresponds to poractant alfa, for which the formulary gives 100 to 200 mg/kg as initial rescue treatment; the preparation should be confirmed before the dose is drawn up [16]. Systematic review evidence supports shorter mechanical ventilation and hospital stays with surfactant in MAS, although mortality is unchanged [22]. International guidance is consistent with this approach: surfactant did not reduce ECMO use in idiopathic PPHN and carries a risk of acute airway obstruction, so it should be reserved for infants with severe parenchymal disease and poor lung recruitment [13].

High-frequency oscillatory ventilation is not generally available outside tertiary centers [1]. Where it is available locally, its use should be discussed with the transport service before starting, particularly when transfer is imminent [1].

Circulation, Blood Pressure, and Inotropes

The aim of circulatory support is to optimize systemic perfusion and ventricular function and, where appropriate, maintain sufficient systemic pressure to reduce right-to-left shunting [2,10]. UK network guidance acknowledges that there is little evidence to identify the optimal inotrope [1]. The response should be judged by improved oxygenation and a narrowing pre- to post-ductal saturation gap, together with lactate and urine output, and not by blood pressure alone [1].

The numerical blood pressure targets differ: the four guidelines give four different figures, with one expressed relative to gestational age rather than as an absolute value. Each is reported with its source in Table 2. This represents genuine variation in practice, so local policy should determine the target. National guidance supports assessing the overall response instead of treating a number in isolation. NICE advises against treating hypotension in preterm babies on the basis of a blood pressure threshold alone and recommends considering other evidence of poor tissue perfusion, with treatment aimed at improving perfusion [11]. Although this recommendation is written for preterm babies, the principle is consistent with UK network guidance for PPHN. International guidance adds an important caution: raising blood pressure to supraphysiological levels solely to drive left-to-right ductal shunting may transiently improve oxygenation but does not lower PVR and should therefore be avoided [13].

Umbilical arterial and venous access should be secured for continuous pressure monitoring and blood gas sampling [1,3]. A trust guideline advises a 10 mL/kg bolus of 0.9% sodium chloride when perfusion is poor or fresh frozen plasma when there is coagulopathy [3]. No volume is stated for the latter; the formulary gives 10-15 mL/kg [16]. Network guidance does not specify a bolus volume but advises against exceeding 20 mL/kg without discussion with the tertiary center and suggests blood or clotting products when indicated [1]. The response should be reassessed before further fluid is given. Hemoglobin thresholds also differ: one network aims for >140 g/L [1], while a second regional guideline aims for >120 g/L [5].

Dopamine and dobutamine are the usual first agents in UK level 2 pathways, reflecting practitioner familiarity, not evidence that either is superior [1]. One network starts dopamine at 10 µg/kg/min and adds dobutamine at 10 µg/kg/min if the response is inadequate. If the response remains inadequate after both have been titrated to 20 µg/kg/min, hydrocortisone 2.5 mg/kg intravenously every six hours is added [1]. A trust guideline likewise starts both agents at 10 µg/kg/min [3]. Neither document presents this sequence as evidence-based. Network guidance instead advises that vasoactive management should be discussed with the transport service and tertiary unit rather than follow a fixed treatment ladder [1].

The doses given here are those the guidelines state, and for the first two agents they differ from national formulary guidance. The British National Formulary for Children gives a starting dose of 5 µg/kg/min for both dopamine and dobutamine, adjusted to a response to a maximum of 20, whereas the documents examined here begin at 10 [16]. Starting at the higher figure brings the infant sooner to the dose above which pulmonary vasoconstriction becomes a concern. The adrenaline and hydrocortisone doses in these guidelines are consistent with the formulary.

Dose-related pulmonary vasoconstriction is an important limitation. Network guidance warns that dopamine above 15 µg/kg/min and adrenaline above 0.5 µg/kg/min can cause pulmonary vasoconstriction, reducing pulmonary blood flow and worsening oxygenation [1]. Dopamine, adrenaline, and dobutamine can also cause marked tachycardia and increase myocardial oxygen demand [1]. Where dopamine has produced a good response, network guidance advises reducing it to 15 µg/kg/min as soon as possible [1]. Adrenaline at 0.1-1.0 µg/kg/min may be used and may be first-line in sepsis or when systemic hypotension is the dominant problem, but any decision to start or increase it should be discussed with the tertiary team [1,3].

Prostaglandin should be started, after discussion with the tertiary or cardiac team, when the diagnosis remains uncertain and echocardiography cannot be obtained [1]. Network guidance notes that it is unlikely to harm and may benefit PPHN by off-loading the right heart; a second regional guideline makes the same point for severe disease and names dinoprostone as the agent [1,5]. The formulary entry for maintaining ductal patency is alprostadil [16], so the preparation and dose should be confirmed against local policy before starting.

The scheme matching the vasoactive agent to the hemodynamic phenotype is set out in Figure 2, presented below with the escalation sequence. This approach is based on physiological reasoning and expert consensus, not randomized comparative evidence. No randomized trial comparing phenotype-guided selection of a vasoactive agent with a standard approach in PPHN was identified in this review.

Sedation, Muscle Relaxation, and Metabolic Control

Sedation has both physiological and humane benefits. Handling and other noxious stimuli can provoke catecholamine surges, increasing PVR and precipitating desaturation [2]. UK guidance advises minimal handling in a quiet environment with adequate analgesia, using intravenous morphine, with fentanyl boluses of 2 µg/kg where additional sedation is needed during ventilation [3,10]. None of the documents examined states a morphine dose. The formulary gives a continuous intravenous infusion of 5 to 20 µg/kg/hour, notes that higher doses are sometimes required, and sets a maximum of 40 µg/kg/hour [16].

Two of the guidelines examined support a low threshold for muscle relaxation when sedation alone does not adequately control oxygenation and ventilation; one specifies a vecuronium infusion of 60-120 µg/kg/hour [3,10], whose upper limit exceeds the formulary range of 60-100 µg/kg/hour [16]. Network guidance adds two cautions: paralysis may adversely affect blood pressure, and neurological status should be documented carefully before it is started [1]. Muscle relaxants may also mask clinical seizures, so cerebral function monitoring should be considered in a paralyzed infant [3]. The wider literature is more cautious, advising against routine paralysis and recommending it only for infants who require high ventilator settings to maintain oxygenation and ventilation within the target range [2]. Taken together, these positions support muscle relaxation as an individualized intervention, not a routine accompaniment to ventilation.

Metabolic control is part of active PPHN treatment, not background care [2,5]. UK guidance advises maintaining normothermia and notes that hypothermia can exacerbate PPHN; when hypoxic-ischemic encephalopathy is also present, the target temperature should be discussed with the tertiary center [1]. It also advises an ionized calcium above 1.0 mmol/L and a magnesium concentration in the upper part of the normal range of 0.7-1.0 mmol/L, correcting if it falls below 0.8 mmol/L, alongside careful monitoring of glucose, electrolytes, and fluid balance [1,3,5].

The transport service guideline goes further, treating magnesium as a therapeutic vasodilator rather than only as an electrolyte to be corrected. It suggests a bolus of 50 mg/kg over 30 minutes where the mean arterial pressure is maintained, repeated, or given as an infusion if effective, with a suggested ceiling serum magnesium of 3 mmol/L [10]. No other document examined recommends this, and it is not a level 2 decision; it is noted because a referring team may see it done by the transport team.

Acidosis is corrected first by improving ventilation and perfusion. Where bicarbonate is used, UK guidance advises maintaining a pH above 7.35 if gas exchange permits and cautions that liberal use may cause hypernatremia and hypokalemia [3,10]. Its use should be discussed with the transport service [3].

iNO: Indications, Dose, and Monitoring

Among pulmonary vasodilator therapies, iNO has the strongest evidence base for improving oxygenation and reducing the need for ECMO in term and near-term infants with PPHN [1,23]. The Cochrane review found that iNO improves oxygenation and reduces the combined outcome of death or ECMO use [23]. This reduction is driven by fewer infants requiring ECMO, not by a reduction in mortality, the number needed to treat to avoid one use of ECMO being five [23]. Outcomes in infants with diaphragmatic hernias were not improved [5,23]. UK network guidance summarizes the evidence similarly and notes that about 40% of infants do not respond to iNO or do not sustain a response [1]; a 2026 review puts the failure rate at about one third [24]. The estimates differ, but both make the same practical point, that a substantial minority will not improve and that the referring team should not read a poor response as a failure of local care.

iNO is not usually available in a level 2 unit and is generally started by the transport team or tertiary center [5]. The guidelines examined differ in the threshold for starting treatment. One network recommends considering iNO at an OI above 15-20 or when OI cannot be calculated, with an oxygen requirement above 60% [1]. A trust guideline and a transport service guideline both use an OI above 15, or a pre- to post-ductal saturation difference above 5% in the absence of congenital heart disease [3,10]. A second regional guideline defines the indication as hypoxemic respiratory failure beyond 34 weeks’ gestation that does not respond to optimal ventilation [5]. All four guidelines specify a starting dose of 20 ppm [1,3,5,10]. International guidance supports this ceiling: doses above 20 ppm do not improve oxygenation or other outcomes and increase the risk of methemoglobinemia [13].

A recurring question for level 2 teams is whether echocardiography must precede iNO. International guidance places greatest emphasis on echocardiographic confirmation of PPHN with extrapulmonary right-to-left shunting [13]. UK guidance does not require local echocardiography before treatment when it cannot be obtained promptly. The transport team may therefore start iNO before departure when the other criteria are met and the clinical picture is consistent with PPHN with persistent hypoxemia despite optimized respiratory support [1,5].

Three principles are particularly important for the referring team. First, recruit the lung before starting iNO, because iNO cannot reach vessels supplying the collapsed lung [5,13]. Second, do not stop iNO abruptly, because withdrawal can cause rebound pulmonary vasospasm and a sharp fall in saturation, even in an infant who appeared not to respond [5,13,23]. Third, monitor methemoglobin and inspired nitrogen dioxide throughout treatment, including during transfer; the monitoring thresholds are given later in this review [5,10]. Weaning, dose adjustment, and decisions about continuing iNO beyond the initial hours belong to the transport team and receiving center.

Milrinone, vasopressin, and sildenafil are not initiated in a level 2 unit under the UK pathways described here. Their place in UK practice, including the reasons for differences between UK and international guidance on sildenafil, is considered after the sections on transfer.

This completes the interventions undertaken by the level 2 unit itself. Table 3 brings these preceding sections together by comparing level 2, tertiary, and ECMO-center capabilities. It also distinguishes within the level 2 setting between interventions that are routine and those that require discussion with the transport or tertiary team. The table therefore provides a framework for the handover that follows, marking the boundary between local stabilization and specialist management.

**Table 3:** Capability by level of care in the UK. ECMO, extracorporeal membrane oxygenation; iNO, inhaled nitric oxide Compiled by the author from the guidance cited in the corresponding sections of this review; principal sources [1,3,5,10].

Capability	Level 2 local neonatal unit	Tertiary unit or ECMO center
Pre- and post-ductal oximetry, arterial gases, serial oxygenation index	Routine: the core of assessment	Routine
Chest radiography and cranial ultrasound	Routine	Routine
Lung ultrasound	Achievable with structured training	Routine
Echocardiography	Variable; use image transfer and tele-echocardiography links to regional cardiology.	Routine, with pediatric cardiology available
Conventional ventilation and surfactant 200 mg/kg	Routine surfactant is reserved for significant parenchymal disease rather than given routinely.	Routine
High-frequency oscillatory ventilation	Not generally available; discuss before starting if transfer is planned	Routine
Dopamine, dobutamine, hydrocortisone	Routine: one network sets this out as a stepwise sequence, which is a local protocol rather than a universal ladder.	Routine
Adrenaline	Available; discuss use and escalation with the tertiary team.	Routine
Prostaglandin	Start locally when duct-dependent disease cannot be excluded, after discussion.	Routine
Milrinone, vasopressin	Only on specialist advice, usually with the transport service or ECMO team	Routine, echocardiography-guided
iNO	Not held; usually started by the transport team	Routine
ECMO	Not available	Designated centers only

Escalation and Transfer

Escalation should be agreed before the infant reaches the point of emergency transfer rather than negotiated during the crisis. UK network guidance recommends discussing cases with the tertiary unit through the regional neonatal transport service at the earliest opportunity, with conference calls involving the transport service and tertiary unit together [1].

The thresholds for discussing ECMO differ between guidelines, and all three positions should be recognized. One network recommends discussing the infant with the ECMO center when the OI exceeds 30 or when there is inadequate response to treatment [1]. A trust guideline advises considering ECMO at an OI above 25 [3]. By contrast, international guidance sets no numerical threshold for this discussion; its criterion is reported alongside the others in Table 2 [13]. Because potential ECMO treatment determines the appropriate transfer destination, this should be clarified before transfer arrangements are finalized, rather than afterward [1]. ECMO itself is restricted to designated centers under the European standard [6].

Two recent sources allow the pathway described here to be compared with practice elsewhere. A published institutional guideline from a North American center, developed cross-disciplinarily for acute stabilization, agrees on the essentials a level 2 unit controls. These are lung recruitment, adequate tissue oxygen delivery, avoidance of harm, and frequent reassessment of a labile process, with management tailored to the underlying diagnosis rather than applied uniformly [9]. It differs in assuming on-site access to iNO and to echocardiography, and the pathway set out here therefore places the corresponding decisions with the transport service and the receiving center. A 2026 review reaches a compatible conclusion on the drugs: iNO remains the only licensed therapy and fails in about a third of infants, while the alternative vasodilators, including phosphodiesterase inhibitors, prostaglandins, and endothelin antagonists, are supported only by observational data [24]. That is the basis on which this review treats milrinone, vasopressin, and sildenafil as decisions for the receiving center rather than for the referring unit.

Both sources have also refined the account given here. PPHN is dynamic and labile, requiring repeated reassessment, not a single assessment, and management should follow the underlying diagnosis rather than treat PPHN as a single entity [9]. That reasoning lies behind the phenotype step, which now precedes any vasoactive drug in Figure 2. The more recent failure rate has been placed alongside the UK estimate so that the referring team's expectation rests on two independent figures, not one [24].

Table 4 lists findings that should prompt an immediate call, while Figure 2 sets out the sequence for the first one to two hours.

**Table 4:** Findings prompting immediate contact with the neonatal transport service. FiO₂, fractional inspired oxygen concentration; PaO₂, arterial partial pressure of oxygen. Compiled by the author from [1,3,10].

Finding	Detail
Severe or persistent hypoxemia	Sustained FiO₂ above 0.5, or PaO₂ persistently below 8 kPa, despite optimized ventilation
Rising oxygenation index	At or above the agreed local trigger and still rising on best conventional support
Widening saturation gap	A pre- to post-ductal difference that is increasing despite treatment
Pulmonary hypertensive crises	Recurrent acute desaturation with handling or suction
Failing circulation	Blood pressure falling despite volume and inotropes at the doses given in the text
Multi-organ hypoperfusion	Rising lactate, oliguria, or other signs of poor perfusion
Diagnostic doubt	Congenital heart disease cannot be excluded and echocardiography is not available

Eligibility is where the documents diverge most sharply, and the difference is clinically important. A trust guideline includes infants born at ≥34 weeks’ gestation or weighing ≥2 kg [3]. A network guideline instead lists gestation <34 weeks and weight <2 kg as separate exclusions, so either criterion alone would exclude an infant [1]. The international guideline takes the same approach, treating both as independent relative contraindications [14]. Thus, an infant born at 33 weeks and weighing 2.2 kg would be eligible under one document but not the others. The criteria applied by the receiving center should therefore be established during the referral call. The remaining criteria are more consistent and are summarized with their sources in Table 2. The international guideline considers gestation <34 weeks a relative rather than absolute contraindication, noting that survival and cerebral infarction rates are worse at 29-33 weeks, although the differences are modest [14].

In practice, delay in referral carries a greater risk than referring an infant who proves ineligible. Early contact, at intubation or when the agreed local OI trigger is reached, allows time for advice, transport preparation, and coordination with the receiving center while transfer remains feasible [1]. Handover should include gestation, birthweight, delivery details, risk factors, resuscitation and Apgar scores, ventilator settings, the latest gas and OI trend with the sampling site stated, infusions, echocardiographic findings, infection screen, maternal blood group, and uploaded imaging.

Waiting for the Transport Team

The referring team will usually care for the infant for several hours between the referral call and the arrival of the transport team. This interval is part of active treatment, not a pause, and the infant may deteriorate during it.

International transport literature supports this approach. Transfer by specialized critical care teams is associated with fewer unplanned events, including airway problems, cardiopulmonary arrest, sustained hypotension, and loss of intravenous access, and with lower mortality [25]. In the UK, neonatal transport is a separate service, not part of pediatric critical care retrieval, so the organizational findings do not transfer directly; the underlying principle does. Early referral allows the benefit of the transport service to begin while the team is being mobilized, rather than when it arrives.

Monitoring should be continuous and include pre- and post-ductal saturations, invasive blood pressure where an arterial line is in place, and cardiac monitoring [5]. Arterial gases should be repeated, and the OI recalculated with each sample so that the trend, rather than a single value, can be communicated to the transport team [1]. A widening pre- to post-ductal saturation gap is an early warning of falling pulmonary blood flow [1].

Handling should be clustered and kept to a minimum because minimal stimulation is part of treatment [2,3]. Sedation and, where used, muscle relaxation should be maintained as continuous infusions before and during transfer, not as intermittent boluses [10]. If paralysis is started, the neurological examination should be documented first [1].

If a pulmonary hypertensive crisis develops before the team arrives, increase inspired oxygen, deepen sedation and analgesia with short-term paralysis where indicated, optimize lung recruitment, support systemic blood pressure, and call for senior help immediately [1]. The transport service should be contacted again rather than waiting for the planned arrival. Network guidance recommends conference calls involving the transport service and receiving tertiary unit, allowing deterioration to alter the management plan and destination where necessary [1].

Practical preparation can reduce delays once the team arrives. Confirm that the tracheal tube is appropriately sized, securely positioned, and without a significant leak; ensure adequate venous and arterial access; and drain any pneumothorax before departure rather than during transfer [10]. Where ECMO is being considered, the cranial ultrasound required before referral should be completed during this interval [1,3,10]. Handover information, clinical notes, maternal details, and imaging should be assembled at the same time.

The family should be involved throughout. The European standard includes parental information as part of the standard of care, with parents informed about PPHN, its treatment, and its short- and long-term consequences, and written information made available [6]. The same standard expects units to have a transfer guideline that staff follow and to ensure that those responsible for PPHN management are appropriately trained [6]. In practice, parents should be told before the transport team arrives why their baby is being transferred, where the baby is going, and what to expect on arrival while being given time with their baby.

The optimal stabilization time at the referring hospital is not known. Shortening this interval, as targeted in adult retrieval, has not been shown to improve neonatal outcomes [25]. Neonatal stabilization times are longer than pediatric times, with reported medians of 80 versus 45 minutes [25]. They are longer still in the infants considered in this review: one study reported a median of 125 minutes for ventilated infants receiving inotropes compared with 63 minutes for infants who were not ventilated [25]. These figures are quoted in the review from an earlier primary study and are not UK data, but the direction is consistent. The referring team should therefore expect the transport team to spend a substantial period stabilizing the infant on the unit.

After handover: management beyond the level 2 unit

This section is included to help the referring team understand the management it is handing over; none of these decisions belongs to the level 2 unit. Areas of divergence between UK and international guidance are summarized in Table 5.

**Table 5:** Differences between the UK guidelines examined and international recommendations. The table sets out the questions on which the UK guidelines examined and international guidance take different positions. It is descriptive: it reports what each document says and does not recommend one over the other. Local network policy takes precedence in every row. Unlike Table 2, which carries one source per row, this table is a deliberate side-by-side comparison: each row is a single question, and the UK and international positions are placed in adjacent columns so that the divergence is visible. Not every document addresses every question, and each cell cites only those that do. Every figure within a cell carries its own citation, so the source of each position remains identifiable. Compiled by the author from the sources cited in each cell. iNO, inhaled nitric oxide; OI, oxygenation index; ECMO, extracorporeal membrane oxygenation; PPHN, persistent pulmonary hypertension of the newborn

Question	Position in the guidelines examined	International position	Practical implication for a level 2 unit
Saturation gap that counts as significant	Two different figures, one treating a gap above 5% as the classic finding and the other requiring above 10% (Table 2)	A difference of 5-10% supports right-to-left ductal shunting but is not diagnostic [6]; echocardiography is required [13].	Use the local figure, but do not treat any gap as diagnostic on its own.
Hyperoxia test	Network guidance considers it no longer useful and potentially harmful through over-oxygenation [1]; a trust guideline retains it where echocardiography is unavailable and states that a short period of 100% oxygen carries no risk in a term infant [3].	Echocardiography is required to establish the diagnosis [13].	Follow local policy; a negative test must not be used to withhold prostaglandin.
Threshold for starting iNO	Lower thresholds, expressed as an oxygenation index in the mid-teens or as an oxygen requirement, and in one case with no number at all (Table 2)	OI above 25, graded Class I, Level A [13]	Those that give a figure start iNO earlier than the graded international recommendation; the decision rests with the transport team.
Threshold for contacting the ECMO center	An oxygenation index well below the ECMO initiation figure, so that the call is made early (Table 2)	An OI above 40 is given as an indication to consider referral [13]; the current ELSO guideline sets no numerical threshold for initiation, that figure coming from the 2017 supplement [14,15].	Those that give a figure trigger the ECMO conversation well before the ECMO threshold is met; the call is a consultation
Sildenafil	Not recommended in acute PPHN where iNO is available [1]; NICE has withdrawn its evidence summary [12].	A reasonable adjunct if refractory to iNO, especially at OI above 25, graded Class IIa [13]; phosphodiesterase inhibitors listed among agents to consider [6]	Do not start sildenafil at level 2 in the UK; understand why international guidance differs.
Milrinone	A reasonable individual-case option, discussed with the tertiary center [1]	Reasonable where there is left ventricular dysfunction, graded Class IIb [13]	Not a level 2 decision; evidence remains limited and confined to short-term endpoints [26,27]
Basis for treating hypotension	Four different numerical targets across the four guidelines (Table 2)	Raising blood pressure to supraphysiological levels solely to drive a ductal shunt should be avoided [13]; national guidance advises against treating on a threshold alone [11].	Judge the response by oxygenation, the saturation gap, lactate, and urine output rather than by the number.
How the gestation and weight criteria for ECMO are combined	Inclusion as 34 weeks or more or 2 kg or more in one document; the two listed as separate exclusions in the other (Table 2)	The two are treated as independent relative contraindications [14].	The same two figures give different answers for a small late-preterm infant; establish the receiving center's criteria during the call.
Magnesium	Correction of a low concentration only, with a normal range of 0.7-1.0 mmol/L and correction below 0.8 [1,3,5]; one transport service additionally gives a therapeutic bolus of 50 mg/kg [10].	Not addressed in the international documents examined	Correct a low magnesium locally; therapeutic magnesium is not a level 2 decision

iNO is weaned cautiously because exogenous nitric oxide suppresses endogenous nitric oxide production, and abrupt withdrawal can cause rebound vasospasm with a sharp fall in saturation, even in infants who showed no initial improvement [5,13,23]. UK guidance advises reducing iNO in 5 ppm steps every one to two hours to 5 ppm, then in 1 ppm steps; international guidance similarly recommends reducing to 1 ppm before stopping [5,13]. It also states that iNO can usually be discontinued within five days and that infants requiring it beyond this period are more likely to have an underlying developmental lung disorder [13]. For the monitoring described earlier, one regional guideline recommends checking methemoglobin every 12 hours and reassessing if it remains above 2%, while transport service guidance recommends titrating iNO within a 5-20 ppm range to keep methemoglobin below 5% [5,10].

iNO should not be used routinely in preterm infants, in whom trials and meta-analyses have not demonstrated the benefit seen at term [5]. All three sources recognize an exception: severe hypoxemia caused by PPHN physiology rather than parenchymal lung disease, particularly after prolonged rupture of membranes before 24 weeks or severe oligohydramnios between 24 and 30 weeks. In such infants, iNO may be considered, and, where possible, antenatal transfer to a center providing iNO should be arranged [5,13]. National guidance advises against routine iNO in preterm infants receiving respiratory support for respiratory distress syndrome unless there are other indications, such as pulmonary hypoplasia or pulmonary hypertension, and notes that use below 34 weeks’ gestation was off-label at the time of publication [11].

Milrinone acts as both an inotrope and a vasodilator and is regarded by UK guidance as a reasonable option in selected cases, particularly when the infant has not responded to iNO. The quoted dose is 500-750 nanograms/kg/min, equivalent to 30-45 µg/kg/hour; the formulary range is wider at the lower end, at 250-750 nanograms/kg/min [16]. The units used for prescribing should be checked against local policy, since both conventions are in use. Because milrinone may lower systemic blood pressure, network guidance suggests 10 mL/kg of 0.9% sodium chloride before starting, with adrenaline alongside, and advises discussion with the tertiary center before it is used in an infant awaiting transfer [1]. International guidance also considers intravenous milrinone an option for selected infants with PPHN and ventricular dysfunction but emphasizes that evidence for efficacy and safety remains limited and that its use should be guided by specialist advice [13].

The evidence base is particularly limited. A Cochrane review identified no randomized or quasi-randomized trials meeting its inclusion criteria and concluded that the efficacy and safety of milrinone in PPHN remain uncertain, recommending its use only within trials [26]. The review has not been updated. A 2024 scoping review of subsequent literature likewise found limited evidence for both milrinone monotherapy and its use as an adjunct to iNO [27]. The studies were heterogeneous and generally assessed short-term outcomes, such as changes in oxygenation and blood pressure, rather than mortality or ECMO requirement [27]. A 2026 review reaches the same conclusion across the class, finding that support for the alternative vasodilators as a group rests on observational studies [24].

Vasopressin, at 0.02-0.1 units/kg/hour, is generally started only after discussion with the ECMO team. Network guidance advises caution above 0.04 units/kg/hour because of the risk of adverse effects [1]. Its use in the newborn is off-label: the product information does not recommend it below 18 years of age, and the formulary gives no neonatal dose, so it is prescribed on specialist advice alone [16].

Sildenafil requires a specific UK interpretation. A Cochrane review found that sildenafil may reduce mortality and improve oxygenation, but the trials were small, the evidence was low to very low quality, and the apparent benefit came from resource-limited settings where iNO was unavailable [28]. UK network guidance therefore considers these findings not directly generalizable to the UK, where iNO is available [1]. Given sildenafil's slow onset and remaining safety and efficacy concerns, it is not recommended for acute PPHN in that network guidance [1].

International guidance takes a different position, considering sildenafil a reasonable adjunctive therapy for infants refractory to iNO, particularly with an OI above 25, with a Class IIa recommendation [13]. The European standard also lists phosphodiesterase inhibitors among the options to consider when the response to iNO is inadequate [6]. The divergence is therefore primarily contextual: both positions draw on the same limited trial evidence, but UK guidance reflects a setting in which iNO is reliably available, and sildenafil is not required as a substitute.

UK regulatory guidance points in the same direction. NICE has withdrawn its evidence summary on sildenafil for pulmonary hypertension in neonates following updated information in the summary of product characteristics concerning off-label use in PPHN [12]. In the UK setting described here, sildenafil should therefore not routinely be initiated by a level 2 unit for acute PPHN without specialist discussion; its use should follow local policy and tertiary specialist advice [1,3].

Refractory hypoxemia and reconsidering the diagnosis

This section describes management at the receiving center, not at level 2, because it concerns infants already receiving iNO. It is included because the referring team may be the first to recognize that the diagnosis needs to be reconsidered. If profound hypoxemia persists despite adequate lung recruitment, hemodynamic optimization, and iNO, unrecognized congenital heart disease, left ventricular dysfunction, and developmental lung disorders should be reconsidered [3,13].

International guidance makes this explicit: in severe PPHN that fails to improve with pulmonary vasodilator therapy, recruitment, or ECMO, evaluation for disorders of lung development, including alveolar capillary dysplasia and genetic surfactant protein disease, is reasonable (Class IIa, Level B) [13]. Echocardiography is particularly important because left ventricular dysfunction can cause pulmonary venous hypertension, which pulmonary vasodilation may worsen [13]. Total anomalous pulmonary venous connection is the cardiac lesion most often missed on early echocardiography; an underfilled left atrium and ventricle should raise suspicion [3].

Alveolar capillary dysplasia with misalignment of the pulmonary veins is a rare, usually fatal disorder associated with abnormalities of the forkhead box F1 (FOXF1) gene and with other congenital malformations [29]. The condition typically presents as PPHN refractory to conventional treatment and should prompt discussion with the tertiary team and family about genetic testing, lung biopsy, and goals of care [3,29].

After the acute phase

Survivors require structured follow-up, with the plan established before discharge. A second regional guideline highlights a substantial risk of long-term neurodevelopmental morbidity after severe disease [5]. Sensorineural hearing loss may present late or progress, so formal audiological assessment with planned re-testing is required, alongside neurodevelopmental surveillance through early childhood [30]. Infants with chronic lung disease or pulmonary hypoplasia also require respiratory follow-up [30]. The European standard identifies long-term, multidisciplinary, and standardized follow-up after discharge as an area requiring further development across Europe [6]. Follow-up plans should be agreed, before discharge and communicated to the family, community team, and general practitioner.

Recommendations

Five changes could improve care before transfer. Three are within a level 2 unit's control; two require wider system coordination.

Agree on Local Thresholds

The six contested thresholds identified in the four guidelines examined are listed in the introduction and detailed in Table 2. Where guidance differs, these thresholds should be incorporated into a locally governed pathway developed with the neonatal network, transport service, receiving tertiary unit, and relevant cardiology, echocardiography, and ECMO teams. Each threshold should be clearly labelled as a trigger for assessment, treatment, consultation, or transfer, with its evidence source and guideline version documented. Where evidence does not support a single threshold, a defined range combined with a clear clinical decision rule may be safer than arbitrary numerical standardization. The European standard expects a unit guideline specifying indications for transfer within a wider national framework [6].

The practical vehicle should be a single PPHN proforma covering recognition, stabilization, and referral, developed jointly by the level 2 unit, transport service, and receiving tertiary center, and used by all three. It would bring the agreed thresholds to the cot side, prompt the observations that matter, provide a structured handover record, and generate data for the audit proposed below. Local proformas were excluded from the guideline sample because they are not published guidance; nevertheless, they are the appropriate instrument for local implementation.

Make the Oxygenation Index Error-Proof

Print the OI formula, including the kilopascal conversion, on the blood gas chart, or use a calculator that requires the units to be declared [1,3]. One of the guidelines examined already directs its readers to a neonatal calculation application for this purpose, and such tools are in wide use in UK units for respiratory indices and for weight-based drug, tracheal tube, and umbilical line calculations [3]. Whichever method is chosen, the units should not be left to memory. Record the index serially and state the sampling site whenever it is communicated.

Train and Connect

The European standard expects appropriate training in PPHN management and 24-hour access to echocardiography, radiology, and laboratory support [6]. For a level 2 unit, this should include structured lung ultrasound training against recognized competency frameworks [20,21], a functioning image-transfer or tele-echocardiography link with regional pediatric cardiology [3], and rehearsal of the referral call itself.

Measure the First Hours

Neonatal transport metrics remain under development, with definitions varying between services, but the UK Neonatal Transport Group already holds data that could support comparison [25]. A shared dataset recording OI at referral, time to referral, and the infant's condition on arrival could show whether early stabilization and transfer processes are improving.

Address the Evidence Gaps

The optimal oxygen saturation target in PPHN remains uncertain [1]. The Cochrane review of milrinone identified no eligible randomized trials and has not been updated [26]. Sildenafil trials were conducted mainly where iNO was unavailable, leaving its role uncertain where iNO is reliably accessible [28]. The European standard identifies similar gaps and calls for a European PPHN database [6].

Artificial intelligence methods are being developed in pulmonary hypertension for earlier detection, risk stratification, and decision support, drawing on echocardiography and other imaging [31]. That work is not specific to the newborn, and none of it should be used to set neonatal treatment thresholds. The tasks it addresses, recognizing a deteriorating trend early and stratifying who needs specialist input, are those a level 2 unit performs manually in the first hours and would be the relevant application if such methods were extended to the newborn.

These changes do not require equipment beyond that already available to a level 2 unit. They require an agreed pathway, an error-proof calculation, a rehearsed referral process, and a way to measure whether the system works.

Limitations

This is a narrative synthesis, not a systematic review, and treatment effects were not pooled. The practice described here is drawn largely from four publicly available UK guidelines, none of which is peer-reviewed. They were selected to represent different types of organizations and different parts of the UK and are presented to illustrate the range of current practice rather than to establish a standard. The sample is not systematic. The guidelines were appraised against the AGREE II domains (Table 1), but by a single appraiser, so this is a structured appraisal rather than a formal AGREE II assessment, and no domain scores are reported. On that basis, all four are treated throughout as consensus documents rather than as evidence-based guidelines. One guideline, from a neonatal transport service, is past its stated review date, as shown in Table 1; it is included as a description of published practice, not as current authority.

National guidance does not fully address this gap. NICE has no guideline specifically on PPHN, having excluded it from scope [8], and the NICE recommendations cited here come from guidance developed for preterm infants [11]. The two international documents used for comparison are also different in nature: one is a graded clinical guideline and the other a set of service standards. Neither was developed specifically for the UK setting.

The transport literature cited concerns neonatal transport generally, not PPHN specifically, and is based predominantly on observational studies from healthcare systems organized differently from those in the UK [25]. Its findings are therefore presented for context and direction rather than as UK benchmarks. Several recommendations also depend on local resources, including echocardiography, high-frequency ventilation, and image transfer. Doses, targets, and eligibility criteria should therefore be checked against the reader's local network guideline and the British National Formulary for Children before use.

Finally, although the bibliographic search strategy is reported in the review methodology, the number of records retrieved and screened at each stage was not recorded prospectively, so the selection of primary literature cannot be reproduced exactly. An exhaustive inventory of eligible UK guidelines was also not compiled; the four examined should therefore be regarded as illustrative rather than representative of all UK practice.

## Conclusions

PPHN may present to units that cannot provide definitive therapies, reflecting the organization and geography of neonatal services, not a failure of care. This makes the first one to two hours critical. During this period, the foundation of management is careful attention to lung volume and oxygenation, to carbon dioxide and pH, to systemic blood pressure and perfusion, to temperature, glucose, calcium, and magnesium, and to sedation. Every one of these is within a level 2 unit's control.

Two practical challenges remain. Guidance for these units is fragmented: the four UK guidelines examined in this review differ across six clinically relevant thresholds, and no NICE guideline addresses PPHN to resolve them. This reflects the nature of the documents themselves: none reports a search strategy, grades its recommendations, or declares a conflict of interest. The divergence is therefore between consensus documents rather than between competing bodies of evidence. Interpretation of escalation thresholds is further complicated by the use of kilopascals for UK blood-gas reporting, while many published thresholds are expressed in mmHg. These are practical inconsistencies that can be addressed through clearer, locally governed pathways. The figures also serve different decisions: the index that prompts the first call to the transport service is not the index that justifies cannulation, and the two are not interchangeable. A level 2 unit should therefore focus on prompt recognition, physiological stabilization, targeted assessment, and early specialist communication, with timely transfer when the infant's clinical needs exceed local capability.
